# Breast imaging and cancer diagnosis during the COVID-19 pandemic: recommendations from the Italian College of Breast Radiologists by SIRM

**DOI:** 10.1007/s11547-020-01254-3

**Published:** 2020-07-13

**Authors:** Federica Pediconi, Francesca Galati, Daniela Bernardi, Paolo Belli, Beniamino Brancato, Massimo Calabrese, Lucia Camera, Luca A. Carbonaro, Francesca Caumo, Paola Clauser, Veronica Girardi, Chiara Iacconi, Laura Martincich, Pietro Panizza, Antonella Petrillo, Simone Schiaffino, Alberto Tagliafico, Rubina M. Trimboli, Chiara Zuiani, Francesco Sardanelli, Stefania Montemezzi

**Affiliations:** 1 Department of Radiological, Oncological and Pathological Sciences, Policlinico Umberto I, Sapienza University of Rome, Viale Regina Elena 324, 00161 Rome, Italy; 2 Breast Imaging and Screening Unit, Department of Radiology, Humanitas Research Hospital, Via Manzoni 56, 20089 Rozzano, Milan Italy; 3 Dipartimento Diagnostica per Immagini, Radioterapia Oncologica ed Ematologia, Fondazione Policlinico Universitario A. Gemelli IRCCS, Rome, Italy; 4 Struttura Complessa di Senologia Clinica, Istituto per lo Studio la Prevenzione e la Rete Oncologica (ISPRO), Via Cosimo il Vecchio, 2, 50139 Florence, Italy; 5 UOC Senologia Diagnostica, IRCCS Ospedale Policlinico San Martino, Genoa, Italy; 6 Department of Pathology and Diagnostics - Radiology Unit, University Hospital of Verona, Verona, Italy; 7 Unit of Radiology, IRCCS Policlinico San Donato, Via Morandi 30, 20097 San Donato Milanese, Milan Italy; 8 Veneto Institute of Oncology IOV - IRCCS, Padua, Italy; 9 Division of Molecular and Gender Imaging, Department of Biomedical Imaging and Image-Guided Therapy, Medical University of Vienna/General Hospital Vienna, Waehringer Guertel 18-20, 1090 Vienna, Austria; 10 Breast Unit Eusoma Certificated, Department of Breast Imaging and Intervention, Istituto clinico S. Anna, Via del Franzone 31, 25127 Brescia, Italy; 11 Azienda USL Toscana Nord Ovest (ATNO), Carrara, Italy; 12 Unit of Radiodiagnostics, Ospedale Cardinal G. Massaia -ASL AT, Via Conte Verde 125, 14100 Asti, Italy; 13 Breast Imaging Unit, Scientific Institute (IRCCS) Ospedale San Raffaele, Via Olgettina 60, 20132 Milan, Italy; 14 Radiology Division, “Istituto Nazionale Tumori IRCCS Fondazione Pascale – IRCCS di Napoli”, Naples, Italy; 15 Department of Health Sciences (DISSAL)- Radiology Section, University of Genova, Via L.B. Alberti 2, 16132 Genoa, Italy; 16 IRCCS - Ospedale Policlinico San Martino, Largo Rosanna Benzi. 10, 16132 Genoa, Italy; 17 Institute of Radiology, University of Udine, Piazzale S. M. della Misericordia 15, 33100 Udine, Italy; 18 Department of Biomedical Sciences for Health, Università degli Studi di Milano, Via Morandi 30, 20097 San Donato Milanese, Milan Italy

**Keywords:** COVID-19, Breast care recommendations, Breast cancer, Priority categories, Personal protective equipment (PPE)

## Abstract

The Italian College of Breast Radiologists by the Italian Society of Medical Radiology (SIRM) provides recommendations for breast care provision and procedural prioritization during COVID-19 pandemic, being aware that medical decisions must be currently taken balancing patient’s individual and community safety: (1) patients having a scheduled or to-be-scheduled appointment for in-depth diagnostic breast imaging or needle biopsy should confirm the appointment or obtain a new one; (2) patients who have suspicious symptoms of breast cancer (in particular: new onset palpable nodule; skin or nipple retraction; orange peel skin; unilateral secretion from the nipple) should request non-deferrable tests at radiology services; (3) asymptomatic women performing annual mammographic follow-up after breast cancer treatment should preferably schedule the appointment within 1 year and 3 months from the previous check, compatibly with the local organizational conditions; (4) asymptomatic women who have not responded to the invitation for screening mammography after the onset of the pandemic or have been informed of the suspension of the screening activity should schedule the check preferably within 3 months from the date of the not performed check, compatibly with local organizational conditions. The Italian College of Breast Radiologists by SIRM recommends precautions to protect both patients and healthcare workers (radiologists, radiographers, nurses, and reception staff) from infection or disease spread on the occasion of breast imaging procedures, particularly mammography, breast ultrasound, breast magnetic resonance imaging, and breast intervention procedures.

## Introduction

At the beginning of February 2020, only three cases of coronavirus disease 2019 (COVID-19) were identified in Italy, all involving people who had recently travelled to China [1]. On February 20, 2020, a severe case of pneumonia due to severe acute respiratory syndrome coronavirus 2 (SARS-CoV-2) was diagnosed in Lombardy, Italy, in a man in his 30s without any history of possible exposure abroad [2]. Within 14 days, many other cases of COVID-19 in the surrounding area were diagnosed, including a substantial number of critically ill patients [3]. On March 9, 2020, the Italian Government implemented extraordinary measures to limit viral transmission restricting movement in the entire national territory to minimize potential contacts with infected people (www.governo.it, 2020). At that time, the total cases in Italy were 10,149 and the deaths more than 630 (www.statista.com, 2020). Since then, a spread of COVID-19 was observed, especially in northern Italy, with a wide severity spectrum, ranging from asymptomatic disease, minor flu-like symptoms to severe pneumopathies or multiorgan failure [3]. Italian case fatality rate [4], at the time of writing, was 14%, one of the highest worldwide (www.statista.com, 2020). Patients who are older and have comorbidities (diabetes, cardiovascular disease, cancer, immunosuppression, obesity, etc.) are the most likely to develop severe forms [5, 6]. In Italy, as of May 13, 2020, there were 221,216 confirmed cases of COVID-19 and 30,911 related deaths (www.statista.com, 2020).

COVID-19 pandemic imposed clinicians a unique set of challenges in the management of women needing periodical screening mammography, imaging, and/or needle biopsy for a suspect breast cancer. Because of the combination of lockdown, need of social distancing, and reduced hospital resources, in many cases the routine outpatient breast imaging activity has been drastically reduced. Therefore, it is crucial to define which women require urgent or non-reschedulable care, can wait reasonable time, or can wait until the pandemic is over [7, 8]. A new paradigm has been introduced by the pandemic facing, on the one hand, with best oncological management, avoiding any potential loss of opportunity [9, 10] and, on the other, extra-protection from the risk of SARS-CoV-2 infection [11].

In this context, the Italian College of Breast Radiologists by the Italian Society of Medical Radiology (SIRM) provides recommendations for breast care provision and procedural prioritization, being aware that medical decisions must be currently taken balancing patient’s individual and community safety. These recommendations are limited to the diagnosis and management of breast malignant disease. Recommendations for oncologic imaging of whole-body staging/restaging are left to general oncologic imaging recommendations (www.esmo.org, 2020; www.asco.org, 2020) [11, 12].

## Priority categories and suggested practice

The response to this pandemic has led to a sudden disruption of routine medical care, including breast imaging workup in asymptomatic population, symptomatic patients, and management of cancer patients [7, 8]. Even early breast cancer can be a fatal disease if left untreated, so adequate surgery combined with appropriate perioperative therapies is essential to improve the outcome [9, 10]. It is well known that early detection contributes to a decrease in specific breast cancer mortality [13–15], and breast cancer outcomes are dependent on timely and high-quality multidisciplinary interventions [14]. A short delay (e.g., 6–12 weeks) should not affect overall outcomes [9, 10], while a diagnostic delay longer than 3 months potentially does [9, 10]. Triage may become necessary to justify which patients should undergo immediate care versus further delay.

In this context, the Italian College of Breast Radiologists by SIRM has defined the following recommendations:

1. patients having a scheduled or to-be-scheduled appointment for in-depth diagnostic breast imaging or needle biopsy should confirm the appointment or obtain a new one;
2. patients who have suspicious symptoms of breast cancer (in particular: new onset palpable nodule; skin or nipple retraction; orange peel skin; unilateral secretion from the nipple) should request non-deferrable tests at radiology services;
3. asymptomatic women performing annual mammographic follow-up after breast cancer treatment should preferably schedule the appointment within 1 year and 3 months from the previous check, compatibly with the local organizational conditions;
4. asymptomatic women who have not responded to the invitation for screening mammography after the onset of the pandemic or have been informed of the suspension of the screening activity should schedule the check preferably within 3 months from the date of the not performed check, compatibly with local organizational conditions.

These recommendations are driven by the common goal to preserve hospital resources for COVID-19 patients by deferring breast imaging procedures without compromising long-term outcomes for individual patients. The demands COVID-19 pandemic will put on healthcare institutions remain unpredictable and will have temporal and geographical variability. Therefore, the risk of disease progression and compromised breast cancer-specific outcomes need to be weighed against viral exposure to patients and staff, considering each individual’s comorbidities and age to predict risk of mortality because of COVID-19. Lastly, these are only recommendations and are not intended to supersede individual physician judgment or institutional policies and guidelines.

All efforts should be made to avoid delayed diagnosis in patients considered at high priority, due to the potential impact on cancer outcome. Suspicious symptoms, clinical or imaging findings are listed below:

1. new onset of a breast palpable nodule [16];
2. new bloody or watery, unilateral, unifocal, nipple discharge [17, 18];
3. orange peel skin [16];
4. skin or nipple retraction [16];
5. search for carcinoma of unknown primary (CUP) syndrome [19];
6. breast abscess, hematoma, or infected seroma [20];
7. clinical suspicion of inflammatory or locally advanced breast cancer [16];
8. suspicion of breast cancer in pregnant women [21];
9. preoperative imaging for local/contralateral staging [22];
10. imaging for monitoring the effect of neoadjuvant therapy [23];
11. recall from screening for further workup [24];
12. indication to breast and/or loco-regional lymph node needle biopsy [20].

Moreover, the multidisciplinary tumor board meetings should be restricted to one professional for each discipline of the core team [25] or, better, online performed involving all the members, for cases requiring multidisciplinary decision (e.g., B3 lesions diagnosed on image-guided breast biopsy, decision to perform or not breast magnetic resonance imaging (MRI) for local and contralateral staging; decision on type and frequency of imaging for monitoring the effect of neoadjuvant therapy, etc.). In the context of current pandemic, multidisciplinary discussions should take into consideration both values and preferences of any individual patient as well as the local epidemiological condition, expected to strongly vary during the next months.

## Personal protective equipment

The Italian College of Breast Radiologists by SIRM promotes the following precautions to protect both patients and healthcare workers (radiologists, radiographers, nurses, and reception staff) from infection or disease spread during breast imaging procedures, particularly mammography, breast ultrasound, breast MRI, and breast intervention procedures, such as biopsy. The close proximity of both the patients’ and healthcare workers’ faces during these imaging procedures raises concern for droplet contamination in addition to blood spatter contamination, which may occur during image-guided interventions and spread by saliva while talking during imaging (https://apps.who.int/, 2020). Everyone involved is invited to follow the indications explained below during breast imaging procedures, avoiding inappropriate, unsolicited, or redundant examinations and being compliant with the established settings.

### Oncological/urgent outpatient, supposed SARS-CoV-2 negative

All patients are considered at risk of SARS-CoV-2 infection. In the waiting room, the patient should be present alone without any accompanying caregiver; close adherence to physical distancing (at least 1 m) is required between each patient. Patients with fever or respiratory symptoms (cough, dyspnoea, cold, sore throat) should be invited to go back home and contact the referring physician. Asymptomatic patients should be invited to sanitize their hands, using the appropriate devices set up at the entrance of the waiting room. Patients wearing gloves should be invited to replace them with new ones provided by the center. Patients must wear a surgical mask (provided by the center if the patient does not wear it at the arrival), and if wearing an FFP2 or FFP3 mask with filter, they should wear a surgical mask over.

All staff having close or physical contact with patients undergoing breast imaging, including interventional procedures (breast biopsy), should wear surgical mask, visor or goggle protection, disposable gown, disposable cap, and disposable gloves. For breast ultrasound, the probe should be covered with a probe cover or glove. All the devices with direct contact to the patient (e.g., ultrasound probe cover, mammographic equipment, magnetic resonance breast coil) should be sanitized with a disinfectant before every patient. The healthcare workers remaining 1 m from the patient may not wear special devices.

### SARS-CoV-2 infection under investigation (patients waiting for swab result)

The patient must wear a surgical mask. All healthcare workers (radiologists, radiographers, and nurses) wear an FFP2 mask, visor or goggle protection, disposable gown, disposable cap, and disposable gloves. The ultrasound cover must be implemented with washable or plastic film on the keyboard and the ultrasound machine, not only for interventional procedures. The ultrasound probe cover must be changed for each patient to prevent potential transmission. Further sanitization of all the devices with which the patient comes into contact (e.g., ultrasound probe and machine, mammographic equipment, and magnetic resonance room) must be performed after undressing at the end procedure to be ready for next dressing.

### Patients positive for SARS-CoV-2 infection

The patient must wear a surgical mask. All healthcare workers (radiologists, radiographers, and nurses) wear an FFP2 or FFP3 mask, visor or goggle protection, disposable waterproof gown, disposable cap, double disposable gloves. Complete coverage of the ultrasound machine and probe must be implemented, not only for interventional procedures. The probe cover must be changed for each patient to prevent transmission of further infection. Further sanitization of all the area in which the patient comes into contact must be performed after undressing at the end procedure to be ready for next dressing. In cases where the patient cannot wear the surgical mask, healthcare workers must wear an FFP3 mask.

## Emotional aspects of cancer patients during COVID-19 pandemic

All the commotion of the COVID-19 pandemic is anxiety-provoking among both patients and practitioners. The aim of these recommendations is to share good clinical practice recommendations in this difficult contest, giving the clinicians the cornerstones to manage patients correctly and safely. Oncological patient is one of the most fragile during the pandemic. The significant psychological impact on oncological patients is compounded by multiple factors: knowledge that the individual is at higher risk of serious complication if infected by SARS-CoV-2, loneliness and isolation as a result of social distancing, and the underlying constant fear of the cancer [26]. Patient psychological well-being needs to be considered and often can be addressed with telemedicine/phone visits. To reduce uncertainty feelings affecting both clinicians and patients, structured recommendations and the adequate psychological support are essential tools for healthcare workers to aid cancer patients to overcome this difficult moment [27, 28].

## Conclusions and future directions

This information should be used to provide radiologists with structured recommendations for breast care in both asymptomatic population and breast cancer patients during the COVID-19 pandemic. In particular, we recommend that diagnostic procedures in symptomatic patients and in patients with a suspicious finding should be confirmed according to local availability and resources. Breast cancer patient’s follow-up and routine breast cancer screening should be performed with a delay of no more than 3 months from the scheduled date, compatibly with the local organizational conditions. All breast imaging procedures should be conducted wearing personal protective equipment precautions, following the measures of social distancing, and the sanitization of equipment and spaces to protect both patients and healthcare workers. However, as the pandemic rapidly evolves, we are increasingly learning about viral transmission and its impact on the healthcare system. Thus, these recommendations may change over time and could need to be updated. It is our hope that this paper will help women, radiologists, and all other clinicians to provide the highest-level care for their patients during this evolving pandemic.
